# Association between friction and wear in diarthrodial joints lacking lubricin

**DOI:** 10.1002/art.22974

**Published:** 2007-11

**Authors:** Gregory D Jay, Jahn R Torres, David K Rhee, Heikki J Helminen, Mika M Hytinnen, Chung-Ja Cha, Khaled Elsaid, Kyung-Suk Kim, Yajun Cui, Matthew L Warman

**Affiliations:** 1 Brown UniversityProvidence, Rhode Island; 2 Case Western Reserve UniversityCleveland, Ohio; 3 University of KuopioKuopio, Finland

## Abstract

**Objective:**

The glycoprotein lubricin (encoded by the gene *Prg4*) is secreted by surface chondrocytes and synovial cells, and has been shown to reduce friction in vitro. In contrast to man-made bearings, mammalian diarthrodial joints must endogenously produce friction-reducing agents. This study was undertaken to investigate whether friction is associated with wear.

**Methods:**

The lubricating ability of synovial fluid (SF) samples from humans with genetic lubricin deficiency was tested in vitro. The coefficient of friction in the knee joints of normal and lubricin-null mice was measured ex vivo; these joints were also studied by light and electron microscopy. Atomic force microscopy was used to image and measure how lubricin reduces friction in vitro.

**Results:**

SF lacking lubricin failed to reduce friction in the boundary mode. Joints of lubricin-null mice showed early wear and higher friction than joints from their wild-type counterparts. Lubricin self-organized and reduced the work of adhesion between apposing asperities.

**Conclusion:**

These data show that friction is coupled with wear at the cartilage surface in vivo. They imply that acquired lubricin degradation occurring in inflammatory joint diseases predisposes the cartilage to damage. Lastly, they suggest that lubricin, or similar biomolecules, will have applications in man-made devices in which reducing friction is essential.

Low friction is a feature of mammalian joints (1,2). Avoiding solid–solid (bare cartilage–cartilage) contact, when mechanical loads can reach 18 MPa and are nominally 5 MPa (3), is important for preserving the longevity of the cartilage surface. The most superficial covering of articular cartilage contains a mucinous glycoprotein (4) that is expressed by superficial zone chondrocytes (5) and synoviocytes (6). This protein, lubricin, provides lubrication in vitro (7,8). Lubricin deficiency in humans causes the autosomal-recessive disorder camptodactyly-arthropathy–coxa vara–pericarditis syndrome (CACP) (9), which is associated with precocious joint failure (10). Mice lacking lubricin also develop precocious joint failure (11) and provide a unique opportunity to study the lubricating behaviors of this glycoprotein in the intact joint. Likewise, synovial fluid (SF) from patients with CACP can be tested for its friction-reducing ability in vitro (7).

Measurement of whole joint friction has been accomplished previously in pendulum systems in which the pivot point of the joint serves as the fulcrum of a pendulum. Classic work by Charnley (1) established that SF provides lubrication in a boundary mode (2), which is independent of articular sliding speed and thus not dependent on joint fluid viscosity. Cartilage lubricated by SF has a very low coefficient of friction (μ) of ∼0.001, which is lower than that of any man-made surface, including Teflon and graphite (12). It has been difficult to study the wear of articular cartilage in the absence of lubricin since enzymatic methods to remove lubricin also destroy other cartilage surface components, and since the complex motions of mammalian joints (13) are not replicable in vitro. Therefore, we developed a pendulum system to study whole-joint friction characteristics in lubricin-mutant (Prg4^−/−^) mice ex vivo. We complemented these studies with in vitro analyses of SF from patients with CACP, using a latex-on-glass bearing system (7) that isolates the conditions of boundary lubrication and replicates lubricating behaviors of cartilage-containing bearings (8). Finally, since lubricating ability can be studied in noncartilaginous bearings such as mica (14) and latex (7), we hypothesized that the atomic force microscope (AFM) could be used to characterize the physicochemical properties of lubricin at the nanoscale, the scale at which boundary lubrication occurs. Our studies show that lubricin reduces friction in vivo as well as in vitro, and suggest that it does so by forming higher-order structures that reduce the work of adhesion between apposing surfaces.

## MATERIALS AND METHODS

### Lubricin-mutant mice

The generation of mice with heterozygous (Prg4^+/−^) and homozygous (Prg4^−/−^) loss-of-function mutations of lubricin has been described previously (11).

### Isolation and harvesting of mouse knee joints

Hind limbs from Prg4^−/−^ and Prg4^+/−^ mice were harvested following carbon dioxide asphyxiation. Hind limbs were prepared for the pendulum apparatus by dissecting away all supporting tendons and musculature with the aid of a dissection microscope. The knee joint synovium was left undisturbed. The other ends of the tibia and femur were also cleaned, enabling their insertion into Plexiglass tubing and fixation with Dermabond. Articular cartilage specimens from knee joints of newborn, 15-day-old, 1-month-old, and 2-month-old Prg4^−/−^ and Prg4^+/−^ mice (2–5 Prg4^−/−^ mice and 2–5 Prg4^+/−^mice per age group) were prepared for microscopy. All studies involving mice were approved by the local institutional animal care and use committees.

### Light and electron microscopy

Knee joints were immersion-fixed in half-strength Karnovsky's fixative with 2% paraformaldehyde and 2.5% glutaraldehyde in 0.1*M* phosphate buffer (pH 7.4) at 4°C for 18 hours (15,16). The specimens were decalcified with 7.5% EDTA in 1% paraformaldehyde and 0.1*M* phosphate buffer (pH 7.4) at 4°C for 10 days (17), followed by rinsing in 0.1*M* cacodylate buffer (pH 7.4). Thin slices cut sagittally across the articular surface from the weight-bearing area of either the femoral or the tibial medial condyle were prepared and trimmed with a razor blade under stereomicroscopic observation. The slices were postfixed in 1% osmium tetroxide buffered to pH 7.4 with 0.1*M* cacodylate buffer, dehydrated in an ascending series of ethanol solutions, and embedded in LX-112 epoxy resin (Ladd Research Industries, Burlington, VT). Vertical ultrathin sections were cut through the whole depth of cartilage with an Ultracut E ultramicrotome (Reichert-Jung, Vienna, Austria) and collected on copper grids covered with Formvar. The specimens were stained for collagen fibrils with 1% tannic acid (Mallinckrodt, Paris, KY) (18) and thereafter with uranyl acetate and lead citrate, using a Leica Reichert Ultrostainer (Leica, Vienna, Austria). The ultrathin sections were examined at 80 kV with a 1200EX transmission electron microscope (JEOL, Tokyo, Japan). Micrographs were digitized with a BioScan CCD camera, model 792 (Gatan, Pleasanton, CA). Additional 1-μm–thick Epon sections prepared for electron microscopy were stained with toluidine blue.

### Joint motion pendulum simulator (modified Stanton pendulum)

**Figure 1:**
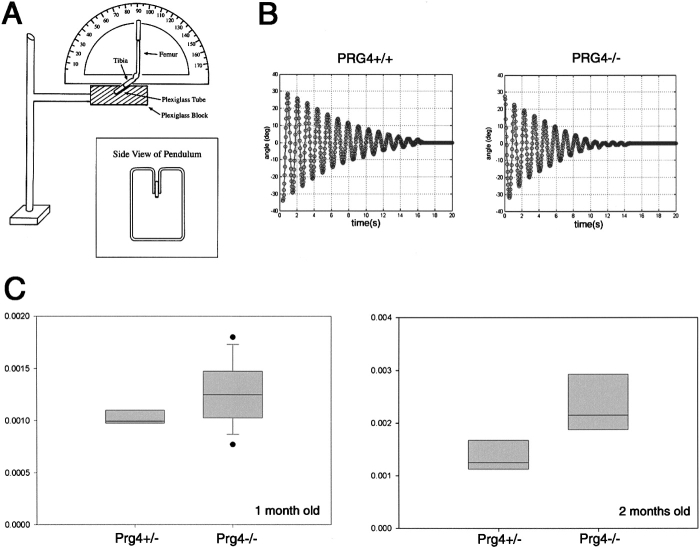
Schema of the pendulum apparatus used to measure whole joint coefficient of friction (μ), and representative data. **A**, Technique of whole-joint measurement using mouse knee joints in which muscles were removed and the joint capsule left intact. The tibia is fixed at a 45° angle so that the knee joint angle is 135° when the femur is held perpendicular. The joint is loaded with 20 gm weight, simulating the weight of an adult mouse standing on 1 limb, with the fulcrum of the pendulum located at the knee joint. The femur is manually deflected from the perpendicular by 30° and allowed to oscillate freely at the knee joint until joint motion stops. **B**, Representative raw data obtained using a modified Moiré encoder technique, measuring the oscillation of knee joints of Prg4^+/+^ and Prg4^−/−^ mice. Deceleration of the pendulum was calculated from the pendulum decay and divided by G, the earth's gravitational constant, to determine the coefficient of friction. Note that the knee of the Prg4^+/+^ mouse oscillated longer and for more cycles than that of the Prg4^−/−^ mouse. **C**, Box plots of μ values in 1-month-old and 2-month-old Prg4^−/−^ and Prg4^+/−^ mice, measured with the pendulum apparatus. Each box represents the 25th to 75th percentiles. Lines inside the boxes represent the median. For 1-month-old Prg4^−/−^ mice, lines outside the box represent the 10th and 90th percentiles, and circles indicate outliers.

The μ value of mouse knee joints was determined by centering the excised joint as the fulcrum of a pendulum (Figure 1A). The tibial end was supported at 45° off the perpendicular by an experimental test stand. The femoral end supported a pendulum, which hung below the excised joint. The mass of the pendulum was 20 gm, roughly the weight of an adult mouse. The pendulum was manually deflected to an angle of 30° off the perpendicular and then released; a CCD camera (GR-DVM90; JVC, Tokyo, Japan) then recorded the motion of the joint. Data were collected at a rate of 30 frames per second until the pendulum came to rest. The angle of the displacement of the joint was measured and analyzed in each frame (Figure 1B), using algorithms developed with MatLab technical computing language software (MathWorks, Natick, MA). The amplitude deflected by the pendulum (in degrees) was plotted against cycle number and time.

The coefficient of friction (μ) was calculated by measuring deceleration of the pendulum (a = −dv/dt) from the image processing analysis. Velocity was determined as (2Gh)^1/2^, where h was the height from where the center of gravity of the pendulum reached the point of maximum velocity at an angle of 0 degrees against the perpendicular. The frictional force (F_f_), acting on the pendulum at the articular surface, was equated to μW, where W is the normal force. Thus, calculation of μ became a ratio of acceleration (a) terms, i.e., μ = F_f_/W = a/G. This calculation presently neglects aerodynamic drag since the projected area of the pendulum is small, neglects viscoelastic dissipation, and assumes gravity (G) to be 9.81 m/sec^2^.

### Synovial fluid collection

Human SF from patients with CACP was recovered as discarded material following diagnostic or therapeutic aspiration. Control (normal) SF (generously provided by Dr. Martin Lotz, Scripps Research Institute, La Jolla, CA) was obtained from allograft cartilage donors. SF aspirates were centrifuged at 12,000*g* at 4°C for 60 minutes to remove cellular debris. The supernatant was decanted and stored at −80°C.

### Purification of human lubricin

Lubricin was purified from human SF as described previously (6).

### Atomic force microscopy

Purified human lubricin was diluted to concentrations of 10, 100, 200, 250 and 300 μg/ml in 0.9% NaCl (physiologic saline). Solutions were deposited on highly ordered pyrolytic graphite (HOPG) substrate to allow for thermodynamic equilibrium to be reached as the lubricin molecules settled on the hydrophobic surface. After 10 minutes the excess solution was removed with a pipette and the remaining fluid was extracted through capillary action, without disturbing the surface. AFM silicon ultralevers for contact mode (Veeco Instruments, Santa Barbara, CA) with spring constants of 0.26 N/m and 0.4 N/m were used to generate a 3 × 3–μm topography image of the surface and subsequently to generate 16 force-versus-distance curves across the HOPG. An instrumentation modification to the AFM that is specific for probing the topography, elasticity, and adhesion of the samples, known as pulse force mode (PFM) (19), was used. The force-versus-distance and adhesive force calculations were performed using MatLab technical computing language software.

### Friction apparatus

The friction apparatus for measuring lubricating ability of SF was an improved version of an instrument developed by McCutchen (2) and modified by Davis et al (8), and has been described previously (7).

### Determination of hyaluronate concentrations

Uronic acid content in SF aspirates was assayed by manual carbazole reaction (20).

### Statistical analysis

Mean values of μ and the corresponding 95% confidence intervals (95% CIs) were determined. Values of μ were compared across genotypes by Student's *t*-test. Box plots were also generated to compare groups. *P* values less than or equal to 0.05 were considered significant.

## RESULTS

### Early postnatal disruption of cartilage surfaces in Prg4^−/−^ mice

**Figure 2:**
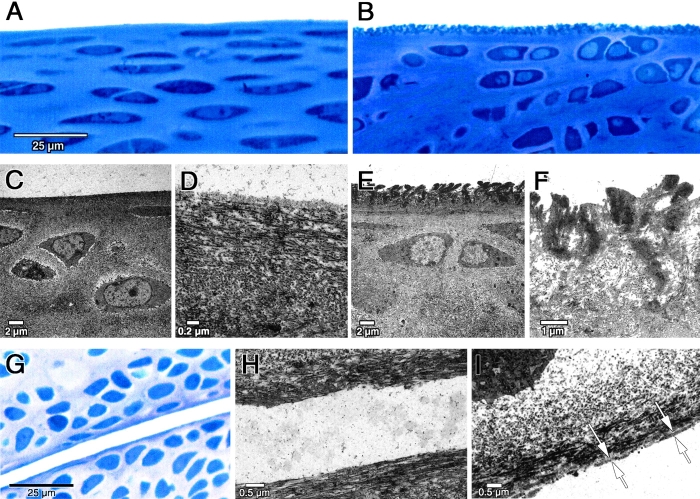
Microscopy of cartilage from heterozygous (Prg4^+/−^) and lubricin-null (Prg4^−/−^) mice. **A** and **B**, Representative photomicrographs of toluidine blue–stained knee joint cartilage sections from 2-week-old Prg4^+/−^ (**A**) and Prg4^−/−^ (**B**) mice, demonstrating differences in surface smoothness. **C** and **D**, Representative electron micrographs of the cartilage surface in sections from 2-week-old Prg4^+/−^ mice, revealing a smooth, uniform surface and a clearly visualized lamina splendens. Note the parallel orientation of collagen fibrils adjacent to the cartilage surface. **E** and **F**, Representative electron micrographs of the cartilage surface in sections from 2-week-old Prg4^−/−^ mice, revealing roughening of the surface, lack of a recognizable lamina splendens, and disruption of the parallel orientation of the collagen fibrils. **G** and **H**, Toluidine blue–stained section (**G**) and electron micrograph (**H**) of the cartilage surface in a newborn Prg4^−/−^ mouse. **I**, Electron micrograph of the cartilage surface in a newborn Prg4^+/−^ mouse. Note that there is no difference in collagen fibril orientation between newborn Prg4^+/−^ and Prg4^−/−^ mice, but only the Prg4^+/−^ mouse has a detectable lamina splendens (**arrows**).

Abnormalities in the light and electron microscopic appearance of the cartilage surface were apparent by 2 weeks of age in lubricin-null mice. Compared with heterozygous (Prg4^+/−^) mice, which do not develop joint disease, Prg4^−/−^ mice exhibited an irregular surface with disruption of the normal, parallel orientation of collagen fibrils (Figures 2A–F). During the newborn period, the cartilage surface was smooth and the collagen fibrils oriented normally in both Prg4^−/−^ and Prg4^+/−^ mice (Figures 2G–I). However, a lamina splendens was present in Prg4^+/−^ mice, but not in Prg4^−/−^ mice (Figures 2H and I).

### Increased friction in the joints of Prg4^−/−^ mice

Knee joints from 1-month-old and 2-month-old Prg4^−/−^ and Prg4^+/−^ mice were studied using the joint motion pendulum simulator (Figure 1A). Younger animals could not be studied due to their small size. The mean μ value in joints from 1-month-old Prg4^−/−^ mice was 0.0012 (95% CI 0.0011–0.0014), which differed significantly (*P* < 0.0001) from that in joints of control animals (0.0009 [95% CI 0.0006–0.0012]). Similarly, the mean μ value in joints from 2-month-old Prg4^−/−^ mice was 0.0023 (95% CI 0.0017–0.0029), compared with 0.0013 (95% CI 0.0011–0.0016) in age-matched controls (*P* < 0.0001) (Figure 1C). Of note, μ values in 2-month-old mice were higher than those in 1-month-old mice of the same genotype (*P* < 0.0001).

### Ability of purified lubricin to self-assemble on HOPG, as demonstrated by AFM

**Figure 3:**
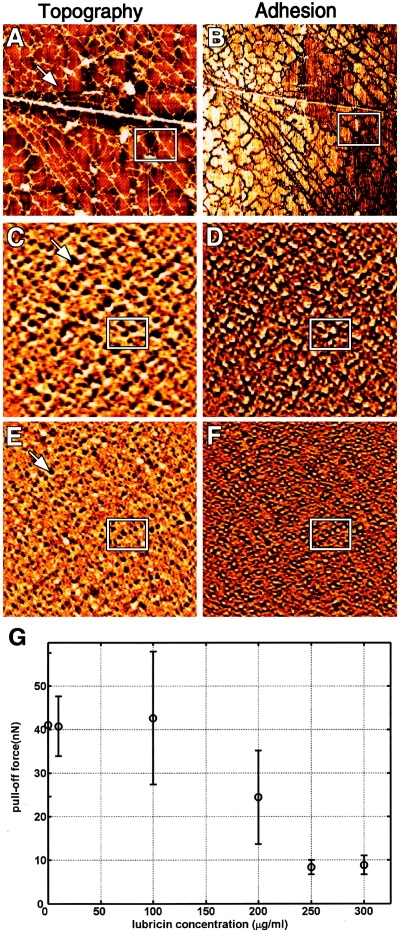
A–F, Atomic force microscopy of lubricin coated onto highly ordered pyrolytic graphite (HOPG). Topographic images (**A**, **C**, and **E**) and adhesion images (**B**, **D**, and **F**) of lubricin networks formed in experiments using lubricin at concentrations of 10 μg/ml (**A** and **B**), 50 μg/ml (**C** and **D**), or 300 μg/ml (**E** and **F**) are shown. In the topographic images, the networks of lubricin molecules are pseudo-colored to appear bright. In the adhesion images, regions of low adhesion are pseudocolored to appear dark. Note that at all concentrations, the lubricin network in the topographic images (bright areas in **A**, **C**, and **E**) corresponds to reduced adhesion (dark areas in **B**, **D**, and **F**). At 10 μg/ml, lubricin formed a loose, mesh-like network structure. At higher concentrations of lubricin (50 μg/ml and 300 μg/ml), the network remained mesh-like, although the mesh size decreased as the concentration increased. **Arrows** indicate an example of openings in the mesh-like network at each concentration. Boxed areas indicate the identical regions in a topographic and corresponding adhesive image, demonstrating the relationship between the presence of lubricin on the HOPG surface and the decrease in adhesion between the surface and the atomic force microscopy (AFM) probe. **G**, AFM force-versus-distance curves obtained in experiments using clean HOPG substrate and substrates coated with lubricin at various concentrations. Note the sigmoidal shape of the curve, which is compatible with lubricin undergoing a phase change when the concentration is ∼250 μg/ml. Values are the mean ± SD.

The pulse force mode of AFM allows for nanoscale visualization of the molecular surface concomitant with measurement of adhesion averaged over the entire area. PFM images from studies using the 10 μg/ml solution of lubricin on HOPG indicated that lubricin could self-assemble into a mesh-like structure (Figure 3A). Sites of low adhesion colocalized with the lubricin molecules in the network (Figure 3B). The use of higher-concentration lubricin solutions (200 μg/ml and 300 μg/ml) resulted in greater coverage of the HOPG surface, coupled with a decrease in the mesh size of the network (Figures 3C and E). Sites of low adhesion continued to colocalize with the lubricin network (Figures 3D and F).

Determination of force-versus-distance curves with clean and lubricin-treated HOPG revealed a sigmoid curve relative to lubricin concentration (Figure 3G). At low lubricin concentrations (10 μg/ml and 100 μg/ml), the adhesive force was the same as that observed with clean HOPG. With further increases in lubricin concentration (200, 250, and 300 μg/ml), the force of adhesion decreased, reaching a nadir with a concentration of 250 μg/ml.

### Lack of lubricating ability of human synovial fluid from patients with CACP

**Figure 4:**
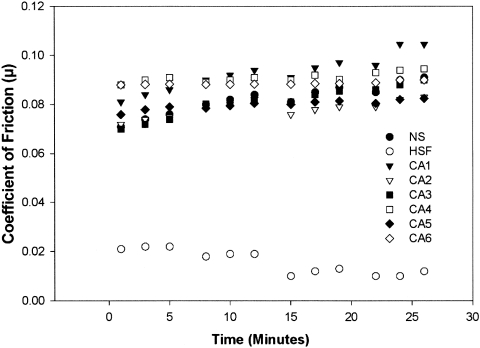
In vitro lubricating abilities of synovial fluid samples. With healthy human synovial fluid (HSF), friction (μ) was reduced to <0.02 and continued to decrease over time, likely due to more uniform covering of the in vitro bearing by the SF. In contrast, SF specimens from 6 individual patients with camptodactyly-arthropathy–coxa vara–pericarditis syndrome (CA1–6) did not reduce friction any more than was observed with normal saline (NS).

Physiologic saline, which has no lubricating ability in the boundary mode, yields a μ value of 0.10. This contrasts with normal human SF, which has lubricating ability and achieves an equilibrium μ value of 0.02. When SF specimens from 6 patients with CACP were tested, all samples failed to lubricate, and μ values were indistinguishable from those obtained with saline alone (Figure 4A). Furthermore, in contrast to normal SF, which exhibited improved lubrication over time as the latex-on-glass bearing became more uniformly covered, there was no evidence of improved lubrication over time with any of the CACP SF samples. Loss of lubrication in CACP SF was not due to loss of hyaluronate, since the hyaluronate concentration in the CACP aspirates averaged 5.45 mg/ml (range 3.82–7.07), which was similar to (but greater than) that in normal human SF (∼2.5 mg/ml).

## DISCUSSION

Our electron microscopy studies revealed absence of a lamina splendens, but an otherwise normal-appearing cartilage surface, in newborn Prg4^−/−^ mice. By 2 weeks of age, the cartilage surface was no longer smooth, and the parallel orientation of collagen fibrils at the surface was disrupted (Figure 2). The incipient damage to the articular surface evident soon after birth suggests that lubricin within the lamina splendens protects the surface from wear by reducing friction. Measurements of whole joint friction (μ) in 1-month-old and 2-month-old Prg4^−/−^ mice support this hypothesis, since they were significantly greater than levels in unaffected littermates (Figure 1). However, the μ value was only twice as high in Prg4^−/−^ mice compared with Prg4^+/−^ animals, possibly due to the ability of pressurized interstitial fluid within cartilage (21,22) to contribute to lubrication via a “weeping” mechanism (2). These data indicate that small increases in friction are capable of causing damage to cartilage surfaces. Therefore, nongenetic disorders, which cause transient deficiency of lubricin as can occur during inflammation, or localized deficiency of lubricin, as can occur following minor trauma, may also predispose to precocious cartilage failure.

It may now be possible to test this hypothesis in animal models of inflammation or localized cartilage damage, since the pendulum apparatus we used enables measurement of whole joint lubrication ex vivo without disturbing normal joint architecture. The μ values we observed are similar to those obtained by Charnley (1), who used a disarticulated, cadaveric, human ankle joint (1). In Charnley's study, swing amplitude versus time showed a linear relationship; this differs from the present results, in which we observed a curvilinear relationship (derived from Figure 1B). Two likely explanations account for this difference. First, we studied the intact knee joint, in which supporting ligaments, synovium, and SF contribute to pendulum damping (23). Second, the knee joint is a roller-slider bearing, since the anterior and posterior cruciate ligaments do not cross the intraarticular space perfectly. The curvilinear relationship we observed is consistent with a sliding movement at high swing amplitude that transitions to a rolling movement at low amplitude. An advantage of the apparatus we utilized is that it retains these normal movements. This is important since the requirements of lubrication change from lowering μ during sliding to reducing adhesion during rolling and static loading. Another advantage of studying the intact joint is that it allows retention of the normal pattern of cartilage deformation that occurs during joint motion. Arthrotripsometers, which load and oscillate annuli of cartilage (24) and other bearing materials, such as latex (2,7,8), are unable to replicate the reciprocating crossed-surface motions (13) or the pressurization of cartilage in its natural state.

To understand how lubricin molecules cover articular cartilage, we visualized purified human lubricin deposited on HOPG, using AFM in the pulsed force mode (19) (Figure 3). Lubricin is able to self-assemble into a mesh-like network and remain surface active, in that areas of lubricin deposition have low adhesion. As the lubricin concentration increases, the mesh openings of the quaternary structure become smaller but the underlying surface remains visible, indicating that the thin film formed by lubricin is nonuniform, at least on HOPG. The AFM images also afford the opportunity to visualize the formation of a network of lubricin molecules that creates an antiadhesive barrier capable of preventing solid–solid contacts, which can otherwise occur in loaded joints. By keeping apposed and flattened articular cartilage asperities separated under load and preventing stick-slip motion, lubricin reduces wear, similar to industrial lubricants such as molybdenum sulfide. The observed sigmoid curve relationship between lubricin concentration and the reduction in work of adhesion of solid interfaces, as measured by PFM, is an indication of the molecules' self-assembly. This supports findings of thermodynamic modeling studies of polymer self-assembly, in which a phase transformation occurs at a critical concentration (25). Our results suggest that lubricin forms a nanofilm within the lamina splendens. This is consistent with previous observations that the cartilage surface has a net negative charge (26), which is removable by serine proteases (27).

To determine whether the genetic absence of lubricin also causes SF to lose its lubricating ability in vitro, we compared control SF with SF from 6 patients with CACP. All CACP SF samples failed to reduce friction (Figure 4). Since CACP SF has normal, and even elevated, levels of hyaluronic acid, we can conclude that hyaluronic acid alone does not function as a joint lubricant. Synergism of the 2 macromolecules in reducing μ has been observed previously (28), and in recent work hyaluronate was shown to reduce friction only when chemically bound to a bearing surface (29). These observations suggest that lubricin, which has a hyaluronate-binding region, may help anchor hyaluronate to articular cartilage.

Human lubricin contains a large central, mucin-like domain, comprising ∼76 repeats of 7 peptide sequences, principally, KEPAPTT, which are sites of extensive *O*-linked β(1–3)Gal-GalNAc (30). This latter posttranslational modification is essential to the protein's lubricating ability (30), conferring steric (31) and possibly hydration force (7), repulsion. Lubricin's binding to hydrophobic surfaces such as latex and HOPG, and its localization to aliphatic–aqueous interfaces (7), are typical of amphipathic polymers. Polysoaps, an amphipathic polymer network, have been shown to possess special properties, including liquid crystalline phases at high concentrations and dense packing of amphiphiles along a polymer backbone, which leads to restricted freedom of conformation and enables steric repulsion (32). Properties of polysoap aggregates have been observed in SF: under conditions of pressure between cartilage and glass, SF adopts a liquid crystalline structure (33). The C-terminus of lubricin may be the origin of surface interactions, including the molecule's ability to interact with articular cartilage (34).

The genetic disorder CACP, in which the lack of lubricin leads to precocious joint failure in the absence of inflammation (10), demonstrates the importance of friction reduction. In the mouse model of CACP (11), in which precocious joint failure also occurs, there is early loss of superficial zone chondrocytes. We speculate that apoptosis of the superficial zone chondrocytes is a consequence of the increased mechanical load that follows a loss of lubricating ability (35). Therefore, acquired deficiency of lubricin or localized disruption of lubricin at the cartilage surface, as can occur in inflammatory joint conditions (36–38) or traumatic joint injury (39), may be the initiating event in more common forms of joint failure. Preventing lubricin degradation may serve to delay joint failure in inflammatory arthritis, and resurfacing damaged cartilage with lubricin (40,41) may delay further damage at those sites. Biomolecules, such as lubricin, may also supplement man-made materials in applications in which low friction and surface protection are required (31).

## AUTHOR CONTRIBUTIONS

Dr. Jay had full access to all of the data in the study and takes responsibility for the integrity of the data and the accuracy of the data analysis.

**Study design.** Jay, Torres, Rhee, Elsaid, Warman.

**Acquisition of data.** Jay, Torres, Rhee, Helminen, Hyttinen, Cha, Elsaid, Cui, Warman.

**Analysis and interpretation of data.** Jay, Torres, Helminen, Hytinnen, Elsaid, Kim, Warman.

**Manuscript preparation.** Jay, Torres, Helminen, Cha, Elsaid, Kim, Warman.

**Statistical analysis.** Jay, Torres.
